# A Systematic Review of Oral Biopsies, Sample Types, and Detection Techniques Applied in Relation to Oral Cancer Detection

**DOI:** 10.3390/biotech11010005

**Published:** 2022-03-02

**Authors:** Guanghuan Yang, Luqi Wei, Benjamin K. S. Thong, Yuanyuan Fu, Io Hong Cheong, Zisis Kozlakidis, Xue Li, Hui Wang, Xiaoguang Li

**Affiliations:** 1 State Key Laboratory of Oncogenes and Related Genes, Centre for Single-Cell Omics, School of Public Health, Shanghai Jiao Tong University School of Medicine, Shanghai 200025, China; yangguanghuan1995@sjtu.edu.cn (G.Y.); weiluqi77@sjtu.edu.cn (L.W.); bksthong@sjtu.edu.cn (B.K.S.T.); yuanyuanfu3@163.com (Y.F.); cheong.iohong@hotmail.com (I.H.C.); lxyolanda@126.com (X.L.); 2 International Agency for Research on Cancer, World Health Organization, 69372 Lyon, France; kozlakidisz@iarc.fr

**Keywords:** oral cancer, biopsy, biospecimen, review

## Abstract

**Background:** Early identification of the stage of oral cancer development can lead to better treatment outcomes and avoid malignant transformation. Therefore, this review aims to provide a comprehensive overview that describes the development of standardized procedures for oral sample collection, characterization, and molecular risk assessment. This can help investigators to choose the appropriate sampling method and downstream analyses for different purposes. **Methods:** This systematic review was conducted according to the PRISMA guidelines. Using both PubMed and Web of Science databases, four independent authors conducted a literature search between 15 and 21 June 2021. We used key search terms to broaden the search for studies. Non-conforming articles were removed using an EndNote-based and manual approach. Reviewers used a designed form to extract data. **Results:** This review included a total of 3574 records, after eliminating duplicate articles and excluding papers that did not meet the inclusion criteria. Finally, 202 articles were included in this review. We summarized the sampling methods, biopsy samples, and downstream analysis. The biopsy techniques were classified into tissue and liquid biopsy. The common sequential analysis of tissue biopsy includes histopathological examination such as H&E or IHC to identify various pathogenic features. Meanwhile, liquid samples such as saliva, blood, and urine are analyzed for the purpose of screening to detect mutations in cancer. Commonly used technologies are PCR, RT-PCR, high-throughput sequencing, and metabolomic analysis. Conclusions: Currently, tissue biopsies provide increased diagnostic value compared to liquid biopsy. However, the minimal invasiveness and convenience of liquid biopsy make it a suitable method for mass screening and eventual clinical adoption. The analysis of samples includes histological and molecular analysis. Metabolite analysis is rising but remains scarce.

## 1. Introduction

Oral cancer is characterized by the presence of a malignant tumor on the lip or in the mouth [1]. Oral and pharyngeal cancers are the sixth most prevalent cancers globally [2,3] and their ranking varies regionally from the first to eleventh place as the most prevalent cancer, with the highest incidence in Southcentral Asia and Melanesia [3,4]. The incidence, mortality, and disability-adjusted life years (DALYs) of oral cancer doubled from 1990–2017 [5]. The age-standardized rates of incidence are about 2.4 per 100,000 while age-standardized disability-adjusted life years are about 64.0 per 100,000 person-years [5]. Asia, in particular, is the region with the highest prevalence and mortality of oral cancer among all continents [6].

Approximately 80% to 90% of malignant lesions in the mouth are recognized as oral squamous cell carcinoma (OSCC) and OSCC accounts for approximately 3% of all cancers globally [7]. The World Health Organization named the precancerous occurrence as oral potentially malignant disorder (OPMD), a condition that may exhibit epithelial dysplasia on histopathologic evaluation [8,9,10]. The anterior part of the tongue, the labial or buccal mucosa, the gingiva, the alveolar muscle, and the palate constitute the potential tissues of origin [11]. The heterogeneous group of conditions associated with OPMD includes leukoplakia, erythroplakia, proliferative verrucous leukoplakia, oral lichen planus, oral submucous fibrosis, palatal lesions, lupus erythematosus, epidermolysis bullosa, and dyskeratosis congenita [10]. Leukoplakia is among the most common OPMDs, with the global prevalence estimated at 1.7–2.7% and the malignant conversion rate at approximately 1.36% [12]. Meta-analysis shows that 7.9% of the OPMD cases turn into a carcinoma with moderate or severe dysplasia [13].

As with most cancer cases, early identification can result in a better treatment outcome. Research shows the 3-year survival rate for the early stage of cancer being 92.2% [14]. The identification of cancer at a later stage causes the 3-year survival rate to fall to 70.3% [14].

However, the diagnosis can be easily missed or delayed as most of the early-stage cancer cases are reported to be asymptomatic.

Due to the difficulty in identifying the site of early lesions, this leads to great difficulties in the early detection and treatment of OPMD and OSCC [15]. Therefore, it is important to confirm the margins of the tumor in order to perform better and more precise lesion removal. Biamonte et al. demonstrated the benefits of combining autofluorescence with a traditional oral examination to determine the surgical margins [16]. Moreover, the advent of precision medicine is anticipated to expedite the development of targeted therapies and screening strategies in the oncology field [17,18]. The application of near-patient diagnostics provides the opportunities to identify early precancerous lesions and intervene in the earlier stage of the disease, treating or delaying the progression of disease via screening and prevention [19]. The understanding of current sampling techniques/biopsies and how these have been used over the recent past can provide a further technical understanding regarding the development of precision medicine techniques for oral cancer. Therefore, this manuscript aims to provide a comprehensive review of oral biopsies, sample types, and detection techniques applied as sampling methods and testing techniques for clinical purposes.

## 2. Methods

This systematic review was conducted according to the PRISMA (Preferred Reporting Items for Systematic Reviews and Meta-Analyses) guidelines [20], including all the published, international, scientific peer-reviewed original research articles.

### 2.1. Databases and Search Strategy

All comprehensive literature recording oral biopsy techniques were taken into account. Using two databases, PubMed and Web of Science, the literature was searched for terms associated with oral cancer (oral cancer, cancer, oral cavity), biopsy (biospecimen, specimen), and molecular assessment (molecular, risk assessment). Each keyword item was bound using the logical word “AND” in the two databases. The detailed search for terms were researched as follows: “oral cancer” AND “biopsy”; “oral cancer” AND “biospecimen”; “oral cancer” AND “specimen”; “molecular” AND “risk assessment” AND “oral cancer”; “cancer” AND “oral cavity” AND “specimen”. These studies were restricted to the human study published on the 21 June 2021. These search strategies were utilized by four authors (**GY, LW, TB, and YF**) to conduct independent searches in the two databases.

### 2.2. Eligibility Criteria and Data Extraction

The results of the databases and manual searches were exported into EndNote. Duplications were removed using EndNote-based methods as reported previously [21], and double-checked manually. Four reviewers (**GY, LW, TB, and YF**) independently screened by following eligibility criteria.

**Figure 1:**
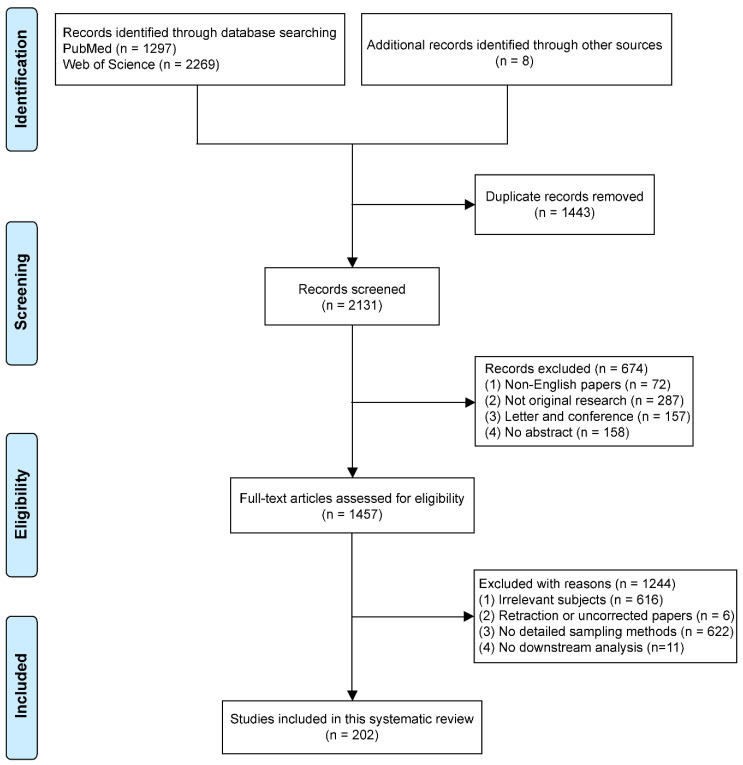
Flow diagram of literature search.

Studies chosen met the following eligibility criteria: (1) All literature searched was relevant to human oral cancer; (2) The samples collected were from human tissue or fluids; (3) Details of the sample collection must be described in the methods available for inclusion; (4) Original studies were eligible for inclusion. However, non-human oral cancers or samples from other species sources were not eligible for inclusion. More importantly, biopsy methods without a detailed description in the literature were not included. Articles which lacked downstream analysis of biopsies were eliminated. Non-English articles, commentaries, and secondary evidence were excluded. The difference of opinion between the reviewers was resolved by a discussion with a third party (**ZK and IC**). The study screening processes are shown in Figure 1.

The four authors (**GY, LW, TB, and YF**) included the publications that met the criteria in Supplementary Table S1. The main items of the article contain the first author, year of publication, patients, type of sample technology, and clinical applications, etc.

## 3. Results

### 3.1. Search Results

A total of 3574 records were included, of which 3566 were found in PubMed and Web of Science. Another eight records were obtained from related professional literature. The PRISMA flow chart for the study selection procedure is shown in Figure 1. After the elimination of duplicates, 2131 papers remained and were screened based on language, article type, and abstract. This resulted in the exclusion of 674 records, including 72 non-English articles, 287 non-original articles, 157 letters/conference reports, and 158 entries only with an abstract and an absence of a full text. The remaining 1457 articles were retrieved for further review. By reading the full text, 1244 articles were excluded because of a lack of direct relevance to the subject, for example only a single mention of oral cancer biopsy (616 papers); retraction (6 papers); manuscripts that utilized biopsies yet did not provide details regarding the sampling methods (622 papers); and no downstream analysis (11 papers). Thus, 202 articles were finally included in this review.

### 3.2. Characteristics of the Included Studies

Supplementary Table S1 describes the characteristics of each included study. Patients were predominantly in the early stages of oral squamous cell carcinoma and potentially malignant disorders. The advantages and limitations of tissue biopsy, liquid biopsy, and brush biopsy are summarized in Table 1. The sampling methods and the techniques used to analyze the biopsy specimens are summarized in Table 2.

The sampling types in the articles can be divided into two main categories depending on the characteristics of the sample: tissue biopsy and liquid biopsy.

### 3.3. Tissue Biopsy 

A tissue biopsy is a method of obtaining soft tissues of the oral cavity or lymph nodes through surgery or special instruments. Traditional tissue biopsy is the most reliable basis for oral cancer diagnosis. There are several methods of tissue biopsy: surgical biopsy, punch biopsy, lymph node biopsy, brush biopsy, and needle aspiration biopsy, implemented through the utilization of various tools such as a scalpel, circular blade, hollow needle, etc. (Supplementary Table S1).

Tissue obtained from the surgical biopsy is made into frozen sections (FS) [22,23] with hematoxylin and eosin (H&E) staining [24,25,26,27,28,29,30,31,32,33,34,35,36,37,38,39,40,41,42,43,44,45,46,47,48,49,50,51,52,53,54,55,56] for pathological analysis. This forms the current gold standard for intraoperative evaluation of tumors. However, study [57] shows that FS requires an extensive waiting time and expensive FS devices. Therefore, the study utilized the multi-staining (MS) [57] method for intraoperative pathological diagnosis. They selected visual inspection acetic acid (VIA) [57] and visual inspection with Lugol’s iodine (VILI) [34,57] in cervical cancer screening due to the high diagnostic accuracy. Other researchers [58] also applied the VILI technique to distinguish epithelial carcinoma and dysplasia from other benign mucosal lesions. Additionally, Takeda et al. applied qPCR and immunofluorescence (IFC) staining to compare normal and cancerous- tissue-related genetic alterations and mitochondrial DNA amounts [59].

SLNB uses frozen section analysis with H&E during surgery to determine the presence of metastasis in cancer. The conventional method is usually paraffin blocks followed by H&E staining [60,61,62,63,64,65,66,67,68,69,70,71,72,73,74,75,76,77,78,79,80,81,82,83,84,85,86,87,88,89,90,91,92,93,94,95,96,97,98,99,100] to examine for possible metastasis and further immunohistochemical (IHC) [83,85,87,88,91,92,96,98,101] analysis for cytokeratin (AE1/AE3) [38,61,63,64,69,72,74,75,77,81,86] to reveal any undetected micro-metastasis if the node was free from tumors. However, the intraoperative pathology of SLNB is only moderately sensitive, and the final pathology examination might require several days. In addition, the SLN identification rate remains relatively low, and the false-negative rate is high. To improve the identification rate, researchers [102] use radioisotopes (RI) and near-infrared (NIR) fluorescence such as indocyanine green (ICG) [89,103] for lymphatic mapping. Besides H&E, these SLNs samples can be cut into 2 mm-thick sections for the preparation of multiple section imprint cytology [104]. These specimens were subsequently stained with May–Grunwald–Giemsa (MGG) and Papanicolaou (PAP) staining [105] for final histopathological diagnosis. Compared to frozen section analysis, imprint cytology requires a shorter time but a lower specificity. Therefore, the selection of techniques was recommended based on the situation. Only a small piece of tissue is required for the pathological assessment of a tissue biopsy. Hence, it is important to identify the margins of the tumor in order to obtain a suitable sample. Autofluorescence can accurately show areas of superficial squamous cell carcinoma of the oral cavity. This helps with further histochemical assessment.

A brush biopsy uses a soft-bristle brush to collect samples from the surface of oral lesions for cytological analysis. This method is usually used as a primary screening method for oral cancer or oral precancerous lesions. Oral brushing samples are then spun down onto slides and stained using the Feulgen-thionin reaction [105,106,107]. Brush biopsies are subsequently stained using PAP staining [28,36,39,43,104,107,108,109,110,111,112,113,114,115,116,117,118,119,120,121,122] or argyrophilic nucleolar organizer region (AgNOR) [32,107,118,121] for conventional cytology (CC) and liquid-based cytology (LBC). For molecular analysis, mass spectrometry [123,124] and DNA-image cytometry (DNA-ICM) [42,125,126,127,128,129] are utilized. DNA-ICM analysis can detect gross alterations of cellular DNA content representing aneuploidy [130]. Due to the lower cost and accessibility of brush biopsies, they are suitable to serve as a robust non-invasive automated oral cancer screening tool for mass screening to promote early oral cancer detection and decrease the number of unnecessary invasive biopsies.

### 3.4. Liquid Biopsy

Liquid biopsies typically use traditional non-invasive methods to collect fluid samples such as saliva, blood, urine, and surgical drainage. When liquid biopsies are performed as a screening tool, all cells have the potential to be analyzed for markers that match the disease. In oncology, fluid samples are analyzed to find mutations in cancer.

A liquid biopsy is a fast, easy method which allows early detection and helps early diagnosis with low risk, minimal pain, and less invasion. It is easily collected and transported for various biomarker detection uses (Table 2). However, the initial histologic diagnosis is still needed for the application of a liquid biopsy. The liquid biopsy has the possibility of “over interpretation” and low sensitivity which may lead to high false results and require greater technical efforts [131].

The sequential analysis of saliva normally includes nucleic acid (PCR [132,133,134,135,136], qPCR [137,138,139,140,141,142,143], ddPCR [122,144], nucleic acid extraction [145], calculation of the DNA integrity index [137] and miRNA expression analysis [141]), protein (Western blot [122,146,147], ELISA [146,148], photometric test [149], proteomic analysis [148,150], etc.), cytological analysis (flow cytometry) [25,125], metabolite (Micro-Raman [151], FT-IR [149,151,152], ATR-FTIR [149], LC-MS/MS [148,153]), and other biochemical analyses (bacterial colony count [146]; immunoreactivity assay [154]). The saliva analysis for the oral cancer biopsy serves as a screening tool for an early diagnosis of oral and oropharyngeal cancer because it has the ability to identify hopefully potential biomarkers for oral carcinoma. In addition, it can be used for the detection of human papilloma virus (HPV) [122,125,138,145,155].

Blood is normally collected for metabolite analysis (metabolite extraction [153], high-performance liquid chromatography analysis [156], GC–MS [148,153], FTIR spectra measurement [152]), biochemical analyses (biochemical estimation [157], chemometric techniques [152], ABC-immunoperoxidase technique [53]) and exosome isolation [153]. It assists diagnosis and the clinical staging of oral carcinoma and serves as the metabolite markers for early detection or diagnosis [131].

Urine and surgical drain fluid can also be collected as a liquid biopsy. The collection of urine is for flow cytometry or high-performance liquid chromatography analysis (Table 2). The evaluation of the surgical drain fluid helps the determination of target levels of different disease outcomes.

## 4. Discussion

Global oral cancer incidence, mortality, and disability-adjusted life years increased by approximately 1.0-fold during 1990–2017 [5]. Obtaining tissue from the oral cavity is an essential first step towards the early identification of potentially malignant lesions and the development of targeted treatments and screening strategies. Therefore, this manuscript aims to provide a comprehensive review of oral biopsies, focusing on the detection techniques applied for different sample types, to select better sampling procedures and detection techniques for oral cancer in clinical diagnosis and precision medicine.

The initial step in these biopsy methods is to analyze the collected samples. In contrast, tissue biopsy obtains solid tissue from the tumor, lesions, or lymph nodes in an invasive manner, whereas liquid biopsy is usually performed using traditional non-invasive or micro-invasive methods to collect liquid samples such as saliva, blood, urine, and surgical drainage fluid (Table 1). Surgical biopsy is a gold standard for diagnosis. The common sequential analysis includes histopathological examination such as H&E [24,25,26,27,95,97,98,99,100] or IHC to identify various pathogenic features [158,159]. Autofluorescence can accurately show areas of superficial squamous cell carcinoma of the oral cavity. This helps with further histochemical assessment [16]. However, it is associated with an increased risk of infection and requires highly skilled professionals.

Meanwhile, the purpose of liquid biopsy is to improve the ability to identify oral cancer and detect prognostic markers of the oral cancer at an early stage. In practice, liquid samples such as saliva, blood, and urine are analyzed for the purpose of screening to detect mutations in cancer. Commonly used technologies are polymerase chain reaction (PCR), real-time polymerase chain reaction (RT-PCR), high throughput sequencing [156], and metabolomic analysis [160].

Liquid biopsy has many advantages over conventional tissue biopsy. In early diagnosis, surgical biopsy is still considered the gold standard, but it causes significant discomfort in the patient. Comparatively, liquid biopsy is a better option for screening and identification of mutations in metastatic cancers and for dynamically following changes that occur during treatment [161,162]. Importantly, in addition to blood, there are other body fluids such as urine, saliva, and surgical drainage fluid samples that can be used for liquid biopsy in oral cancer [131,163,164].

The downstream analyses of liquid biopsies commonly examine circulating tumor cells (CTCs), circulating tumor DNA (ctDNA), circulating tumor RNA (ctRNA), proteins, and exosomes [153,165,166]. CTCs are released from the primary tumor or distant metastatic areas of the tumor cells into the bloodstream and share most of the mutational profile with the primary tumor [167]. Therefore, they could be used as a predictor for the recurrence of oral cancer [168,169]. Circulating cell-free DNA (cfDNA) also has the potential to detect malignancies [170,171], as do exosomes [172,173]. However, such techniques still require overcoming the low detection rates in the early stages of oral cancer, and further standardization is needed prior to any clinical application [174,175].

As liquid biopsy analyzes the cfDNA of cancer patients whose tumor cells are excreted into the bloodstream, it can be expected to provide a complete picture of all the mutations found in all tumors [176]. However, this means that not all mutations are equally well captured by cfDNA [177,178]. Liquid biopsy may not detect a tumor mutation in situ. There is also a lack of competence in the qualitative assessment of tumors. Therefore, performing tumor biopsy at the time of cancer diagnosis can provide important pathological information and the ability to assess biomarkers that do not involve DNA alterations [179].

Similarly, salivary biomarkers have the potential to be utilized as adjunct diagnostic methods. In the case of oral cancer, salivary mitochondrial DNA has been proved to be a prognostic marker [180,181]. In addition, the development of high-throughput sequencing technology is very promising for the application of liquid biopsy to a large-scale clinical screening [131,161]. Liquid biopsy is an innovative, promising approach for cancer detection and therapy. Although liquid biopsies revolutionized oncology, they are not ready to completely replace tissue biopsy [182].

In contrast to liquid biopsy methodologies, the brush biopsy has been used as a common screening technique for oral cancer and does not require strict pre-procedure preparation. It collects cells from the deeper layers of the oral mucosa with minimal pain and bleeding [183]. A brush biopsy is a painless non-invasive method that involves the application of a brush to collect oral cancer specimens [184].

The biopsy can easily rule out the atypical hyperplasia and cancer under limited conditions [185]. The results are reported as negative, atypical, and positive. This is a viable chair-side method for mass screening and initial judgment (decision making) before performing a painful biopsy. The test requires basal cells, thus is likely to result in minor, localized bleeding. However, the keratinization or deep lesions might underrepresent the samples and result in a false-negative outcome [186].

Currently, traditional exfoliative cytology techniques are gradually transforming into computerized cell morphometry through DNA index measurements, micronucleus analysis, and the evaluation of nucleated tissue zones. The addition of molecular methods, such as immunohistochemistry [34,187], cytological analysis [28,36,39,109,125,188], and fluorescence-activated cell scanning [189] can significantly improve efficiency and revolutionize the technique.

Liquid and tissue biopsy techniques are used as two different types of examination methods and functions. More research and advances are necessary to make a decisive choice of superior biopsy. More research is needed on the effectiveness of liquid biopsy as a promising technique for the clinical diagnosis of oral cancer and for guiding treatment.

## 5. Limitations

Non-English articles were excluded from this review, which may have led to the absence of some research in this area. In addition, none of the pre-print depositions were considered, and that might provide a reduced ability to detect any latest developments in the field. A large number of articles were excluded as those articles provided insufficient or unspecific information on the type of biopsy methods used and downstream analysis. Beyond the need for emphasizing methodological accuracy and completeness, this may have resulted in a lack of comprehensive evaluation of trends in the clinical application of biopsy methods.

## 6. Conclusions

The main modalities for oral cancer biopsy remain the invasive ones as they form the current clinical gold standard (such as surgical biopsy, lymph node biopsy, puncture biopsy, etc.). However, increasingly non-invasive methods are applied (such as liquid biopsy and brush biopsy) and the anticipation is that the clinical adoption of the latter will continue to increase. Furthermore, the relationship between oral cancer biopsy samples and downstream analyses remains complex, with the need for harmonization and standardization for a number of downstream molecular methods.

## Figures and Tables

**Table 1:** Advantages and limitations of sampling for oral cancer.

Categories	Type of Sampling	Advantages	Limitations
Tissue biopsy	● Surgical biopsy ● Punch biopsy ● Needle aspiration biopsy ● Sentinel lymph node biopsy ● Brush biopsy	● Suitable for localized lesion ● Most accurate for assessment ● A small fragment of tissue is sufficient	● Invasive ● Bleeding ● Risk of infection ● Time intensive ● Delayed diagnosis ● Require greater technical efforts
Liquid biopsy	Blood, saliva, urine, and surgical drain fluid collection	● Non-invasive or less invasive ● No/less bleeding ● Easily collected ● Easily transported ● Detect various biomarkers ● Tracking dynamic changes ● To facilitate repeat collections	● Inaccurate ● Low sensitivity ● Need an initial pathological diagnosis

**Table 2:** Summary of downstream applications of oral cancer biopsy methods included in the literature.

Categories	Type of Sample	Techniques
Tissue biopsy	● Oral lesions ● Sentinel lymph node	H&E; IHC; VIA; SSOCT; Specimen-driven intraoperative assessment; RT-PCR; Lugol’s iodine staining; ICC; Frozen section; RT-PCR; Immunofluorescence staining; RI; ICG; Routine pathologic diagnosis; p53 IHC; PCR; Direct visualization of the oral tissue autofluorescence; Methylene blue and microendoscope; Ultrasound scanning and histology; Molecular-specific fluorescent contrast; Agent and single-wavelength spectroscopy; PAS; PCNA; Trypan blue exclusion assay; Cytological smears; DNA extraction; Hybridization reconstruction test; Probe removal and re-use of DNA blot; Saline extract; FISH analysis; PAP; Melanoma-associated antigens; Dielectrophoretic method; Feulgen staining; The electrochemical telomerase assay; Frozen section analysis; Serial sectioning; Near-infrared imaging with ICG; Gamma-ray probe; Pan-cytokeratin antibody (AE 1/3); Anti-cytokeratin 22 immunohistochemistry; MGG
Liquid biopsy	● Saliva	Elisa and dot blot tests; Bacterial colony count; Flow cytometry; Total protein estimation by photometric test; ATR-FTIR; PAP; MGG; Micro-Raman; FT-IR; Western blot analysis; ddPCR; Proteomic analysis (LC-MS/MS, ELISA); qPCR; Calculation of the DNA integrity index; RT-qPCR; Immunoreactivity assay; Metabolomic analysis; Nucleic acid extraction; HPV genotyping; Microarray platform; Western blotting and reporter gene assays; 5,5′dithiobis, 2-nitrobenzoic acid (DTNB/Ellman’s reagent); Quantitative methylation-specific PCR; Biochemical analysis of saliva; Tolonium chloride staining;
	● Urine	Bacterial colony count; Flow cytometry; High-performance liquid chromatography analysis; PCR and laboratory analyses (homocysteine and methylenetetrahydrofolate reductase)
	● Blood	Exosome isolation; Metabolite extraction; GC–MS; ddPCR; FTIR spectra measurement; Colorimetry; Biochemical estimation and ninhydrin method; High-performance liquid chromatography analysis; PCR; Laboratory analyses (homocysteine and methylenetetrahydrofolate reductase); RT-PCR; ABC-immunoperoxidase technique
	● Surgical drain fluid	Electrochemiluminescence; Patterned array; Multiplex technology

Abbreviations: H&E: hematoxylin and eosin staining; VIA: visual inspection acetic acid; VILI: visual inspection with Lugol’s iodine; SSOCT: swept-source optical coherence tomography; PAP: Papanicolaou stain; MGG: May–Grunwald–Giemsa; ddPCR: droplet digital polymerase chain reaction; IHC: immunohistochemistry; RT-PCR: real-time polymerase chain reaction; ICC: immunocytochemistry; ICG: indocyanine green; RI: radioisotope; PAS: periodic acid–Schiff; AgNOR: silver-stained nucleolar organizer regions; PCNA: proliferating cell nuclear antigen; MALDI-ToF MS: matrix-assisted laser desorption/ionization time-of-flight mass spectrometry; FISH: Fluorescence in situ hybridization; GC-MS: gas chromatography–mass spectrometry; FTIR: Fourier-transform infrared spectroscopy.

## Data Availability

Not applicable.

## Supplementary Materials

The following supporting information can be downloaded at: https://www.mdpi.com/article/10.3390/biotech11010005/s1, Table S1: Summary characteristics of included studies [190,191,192,193,194,195,196,197,198,199,200,201,202,203,204,205,206,207,208,209,210,211,212,213,214,215,216,217,218,219,220,221,222,223,224,225,226,227,228,229,230,231,232,233,234,235,236,237,238,239,240,241,242,243,244,245,246,247,248,249,250,251].
