# Supplementary material for: A Systematic Review of Oral Biopsies, Sample Types, and Detection Techniques Applied in Relation to Oral Cancer Detection
Source: BioTech (Basel). 2022 Mar 2;11(1):5. doi: 10.3390/biotech11010005 (PMC9245907; doi:10.3390/biotech11010005)
Supplement: Supplementary file 1 [file biotech-11-00005-s001.zip › biotech-1580883-supplementary.pdf]

Table S1. Summary characteristics of included studies. [a] (n = 202)

Reference	Country [b]	Patients	Number of cases/total individuals [c]	Staging (number)	Specific Method of Sampling (Type of Sample)	Techniques	Clinical applications
Xiao et al. 2021 [146]	China	OSCC	20/30	NA	Liquid biopsy: 1) saliva; 2) urine	1) Elisa and dot blot tests; Bacterial colony count; Flow cytometry 2) Flow cytometry	Oral cancer screening
Sugiyama et al. 2021 [99]	Japanese	OC	32	T1(12); T2(20)	SLNB: sentinel lymph node	H&E	Pathological SLN diagnosis
Shaikh et al. 2021 [149]	India	OSMF	15/20	I (9); II (6)	Liquid biopsy: saliva	Total protein estimation by photometric test; ATR-FTIR	As a screening tool for an early diagnosis OSMF
Shah et al. 2021 [190]	India	OPMD	30	NA	Solid biopsy*: oral lesions**	Sediment cytology	Preliminary diagnosis
Putri et al. 2021 [57]	Indonesia	OC	26/54	NA	Surgical biopsy: oral lesions	VIA; VILI	Histopathology examination
Park et al. 2021 [100]	Korea	TSCC	91/211	T1/T2	SLNB: sentinel lymph node	Frozen section analysis; H&E	Pathologic examination
Parfenova et al. 2021 [130]	Canada	OC	92/214	NA	Brush biopsy: suspicious clinical lesion	DNA- ICM	Non-invasive screening of high-grade oral lesions
Obade et al. 2021 [24]	Malaya	OSCC	14/44	NA	Surgical biopsy: oral lesions	1) SCOTT 2) H&E	1) To examine the ability of OCT to differentiate ex vivo epithelial structure of benign disorders, dysplastic, and OSCC in comparison with the structure of normal marginal mucosa of oral biopsies. 2) Histopathological diagnosis
Mahieu et al. 2021 [101]	Netherlands	OSCC	20	cT1-2N0M0	SLNB: sentinel lymph node	Serial-sectioning; IHC	Histopathology
Mahieu et al. 2021 [98]	Netherlands	OSCC	816	cT1-2N0	SLNB: sentinel lymph node	IHC; H&E	Histopathology
Mada et al. 2021 [25]	India	OC	16/24	T2(8); T3(1); T4(7)	1) Surgical biopsy: oral lesions 2] Liquid biopsy: saliva	1) H&E 2) PAP; MGG	1) Histopathological diagnosis 2) Cytological analysis
Li et al. 2021 [191]	China	OPMD	810	NA	1) Brush biopsy: oral lesion 2) Surgical biopsy: oral lesions	1) DNA-ICM 2) NA	1) Oral cancer screening; Establish risk model based on DNA aneuploidy for the detection of oral cancer; Prognostic marker 2) Histopathological examination
Giovannacci et al. 2021 [26]	Italy	OPMD	60	NA	Surgical biopsy: oral lesions	H&E	Histopathological diagnosis
Gaida et al. 2021 [108]	Germany	Clearly visible oral lesions	2018	NA	Brush biopsy: oral lesion	PAP	Cytological analysis
Galíndez et al. 2021 [192]	Argentina	OSCC/OPDM	140	NA	Brush biopsy: oral lesion	PAP; DNA extraction; Genotyping	To improve prevention and early diagnosis.
Falamas et al. 2021 [151]	Romania	OSCC	19/32	II(5); III(2); IV(12)	Liquid biopsy:saliva	Micro-Raman; FT-IR	Diagnosis of oral and oropharyngeal cancer
den Toom et al. 2021 [97]	Netherlands	OSCC	20	cT1-2N0M0	SLNB:sentinel lymph node	H&E	Histopathological diagnosis

de Koning et al. 2021 [27]	Nether-lands	SCCT	91	NA	Tissue biopsy: oral lesion	H&E	Histopathological examination
de Kerangal et al. 2021 [95]	France	OSCC	94	T1-T2cN0	SLNB: sentinel lymph node	Frozen section; H&E	Histopathological examination
Boeve et al. 2021 [96]	Nether-lands	OSCC	91	cT1-2N0/ pT1-2	SLNB: sentinel lymph node	1) H&E<br>2) IHC	1) Tissue microarray construction<br>2) Identification of tumor markers
Aaboubout et al. 2021 [193]	Nether-lands	OCSCC	415	NA	Surgical biopsy: oral lesions	Specimen-driven intraoperative as-sessment	The frequency, type and results of intraoperative assess-ment of resection margins were analyzed.
Yokoyama et al.2020 [94]	Japan	OC	11	NA	SLNB: sentinel lymph node	Frozen section; H&E	Histopathological examination
Wojakowska et al.2020 [153]	Poland	OSCC	10/20	NA	Liquid biopsy: blood	Exosome isolation; Metabolite Ex-traction; GC–MS	Metabolites that differentiated cancer and control sam-ples.
Wei et al. 2020 [194]	USA	OC	NA	NA	1) CEUS-CNB: suspicious le-sions<br>2) Surgical biopsy: suspicious lesions	NA	Histopathological examination
Wang et al. 2020 [122]	UK	OPC	10	NA	Liquid biopsy: saliva	Western Blot Analysis; ddPCR	Detecting HPV16
Vigili et al. 2020 [93]	Italy	OSCC	48	T1–T2N0	SLNB: sentinel lymph node	H&E	Histopathological examination
Velleuer et al. 2020 [109]	Germany	OC	713	NA	Brush biopsy: oral lesion	1) PAP<br>2) DNA-ICM	1) Cytological investigation;<br>2) DNA ploidy analysis
Smits et al. 2020 [195]	Nether-lands	OCSCC	174	NA	Surgical biopsy: oral lesions	Intraoperative assessment	Identification of excision margins; Histopathological evaluation
Sivadasan et al. 2020 [148]	India	OSCC	NA	NA	Liquid biopsy: saliva	Proteomic analysis; LC-MS/MS; ELISA	Identification of candidate biomarkers for the early de-tection of oral squamous carcinoma.
Sandhya et al. 2020 [196]	India	OSCC	112	NA	USG-FNAC: oral lesions	NA	Cytopathological evaluation; Histopathological exami-nation
Rathod et al. 2020 [92]	India	OC	20	I/II	SLNB: sentinel lymph node	IHC; H&E	Histopathological examination
Li et al. 2020 [197]	China	OPMD	401	NA	Brush biopsy: oral lesion	DNA-ICM	Detection of dysplasia and/or cancer in OPMD
Kim et al. 2020 [89]	Korea	OC	9	T1/T2	ICG-Guided SLNB: sentinel lymph node	H&E	Histopathological examination
Ishiguro et al. 2020 [90]	Japan	Tongue cancer	27	NA	SLNB: sentinel lymph node	H&E	Pathological examination
Ines Criscuolo et al. 2020 [125]	Argentina	OSCG	72/144	NA	1) Brush biopsy: oral lesion<br>2) Liquid biopsy: saliva	PCR	HPV detection; Cytological analysis
Hernando et al.2020 [91]	Spain	OSCC	12	T1/T2	SLNB: sentinel lymph node	IHC; H&E	Histopathological examination
Hasegawa et al. 2020 [198]	Japan	OSCC	141	II(59); III(34); IV(48)	Surgical biopsy: oral lesions	NA	Histopathological examination
Durham et al. 2020 [199]	Canada	OSCC	457	T1/T2	Surgical biopsy: oral lesions	NA	Histopathological examination

Crimi et al. 2020 [144]	Italy	OC	10/20	NA	Liquid biopsy: blood	ddPCR	Bioinformatics Analyses
Bhatia et al. 2020 [28]	India	OSMF	24	II(8); III(10); IV(6)	1) MBB: buccal mucosa<br>2) Punch biopsy: buccal mucosa	1) PAP<br>2) H&E	1) LBC<br>2) Histopathological examination
Azab et al. 2020 [137]	Egypt	OSCC/ OPMD	63/93	NA	Liquid biopsy: saliva	qPCR; Calculation of the DNA integrity index	Diagnostic abilities
Aggarwal et al. 2020 [200]	India	OPMD	200	NA	Surgical biopsy: oral lesions	NA	Histopathological examination
Vishnoi et al. 2019 [88]	India	OSCC	94	cT1/T2, N0	SLNB: sentinel lymph node	IHC; H&E	Histopathological examination
Tang et al. 2019 [138]	Australia	OPC	127	NA	Liquid biopsy: saliva	qPCR	Detecting HPV16
Sun et al. 2019 [201]	China	OSCC/ OPMD	269	NA	Brush biopsy: oral lesion	1) DNA-ICM<br>2) H&E	1) Assessment of the cancerization risk in OPMD patients;<br>2) Histopathological examination
Raman et al. 2019 [110]	India	OSCC	150	NA	Biopsy with spatula: buccal mucosa	1) PAP<br>2) MGP	1) Cytological diagnosis<br>1) Cytomorphometry
Muraki et al. 2019 [29]	Japan	OSCC	89	NA	Surgical biopsy: oral lesions	IHC; H&E	Histopathological examination
Mishra et al. 2019 [202]	India	OSMF	15/30	NA	Surgical biopsy: oral lesions	IHC	Detection of human telomerase reverse transcriptase (hTERT) protein telomerase expression
Kujan et al. 2019 [188]	Australian	OSCC/ OLK	55	NA	1) Brush biopsy: oral lesion<br>2) Surgical biopsy: oral lesions	1) OLBC<br>2) IHC	A combined index score of OLBC grading and protein expression was calculated.
Gnanatheepam et al. 2019 [203]	India	OC	88/101	NA	Biopsy with scalpel: oral lesion	SLS	Diagnosis of oral cancer.
Deuerling et al. 2019 [110]	Germany	OSCC	1359	NA	Brush biopsy: oral lesion	PAP	Cytological diagnosis
Riese et al. 2018 [87]	Germany	OC	36	cN0/ cM0	SLNB: sentinel lymph node	1) H&E<br>2) IHC	1) Histopathological examination<br>2) Detection of anti-pancytokeratin epithelial markers
Rai et al. 2018 [152]	India	OSMF	30/60	NA	Liquid biopsy: blood	1) FTIR Spectra Measurement<br>2) Chemometric Techniques	Diagnostic prediction and prognostication of OSF
Pereira et al. 2018 [112]	India	OSCC/ OPMD	20/30	NA	1) Oral rinse-based technique: expectorate<br>2) Brush biopsy: oral lesion	PAP	Cytological diagnosis
Kiran et al. 2018 [113]	India	OSCC/ED	30/60	NA	Biopsy with spatula: buccal mucosa	PAP	Cytological diagnosis
Eisenberg et al. 2018 [155]	USA	Oropharyngeal cancer	395	NA	Liquid biopsy: saliva	DNA analysis	Detection of any HPV
Alsarraf et al. 2018 [114]	Australia	OSCC/ OPMD	10	NA	1) Brush biopsy: oral lesion<br>2) Surgical biopsy: oral lesions	1) PAP<br>2) NA	1) Cytology diagnosis<br>2) Histopathological examination
Al-Dam et al. 2018 [204]	Germany	OC	20	Ct1/cT2N0	SLNB: sentinel lymph node	NA	Histopathological examination

Zarate et al. 2017 [126]	Argentina	OPMD/OC	93/111	NA	Brush biopsy: oral lesion	PCR	P53 genotyping
Tang et al. 2017 [139]	Australia	HNSCC	68/110	I(3); II(1); III(11); IV(49)	Liquid biopsy: saliva	RT-qPCR	To investigate the mRNA expression profiles of CKs
Rezazadeh et al. 2017 [205]	Iran	OSCC	28/48	NA	Brush biopsy: oral lesion	ICC	Identifying cytological biomarkers
Remmerbach et al. 2017 [115]	Germany	OSCC	81/133	NA	Brush biopsy: oral lesion	PAP	LBC
Quang et al. 2017 [30]	Mexico	OC	100	NA	Punch biopsy: oral lesion	1) H&E<br>2) IHC	1) Histopathological examination<br>2) Detection of markers
Perera et al. 2017 [206]	Australia	OSCC/ FEP	52	NA	Surgical biopsy: oral lesions	1) RT-PCR<br>2) HTS	To characterize the mycobiome
Peisker et al. 2017 [154]	Germany	OSCC	30/60	NA	Liquid biopsy: saliva	Immunoreactivity assay	Cancer detection
Parakh et al. 2017 [207]	India	OPMD	40	NA	Punch biopsy: oral lesion	NA	Histopathological examination
Nanami et al. 2017 [208]	Japan	OSCC/ OPMD	62	NA	Surgical biopsy: oral lesions	NA	Histopathological examination
Nair et al. 2017 [209]	India	OSCC	40/80	NA	Liquid biopsy: blood	Colorimetry	Determination of BChE levels
Miura et al. 2017 [86]	Japan	OSCC	57	N0	SLNB: sentinel lymph node	1) H&E<br>2) AE1/3 cytokeratin stain	1) Histopathological examination<br>2) Final postoperative diagnosis
Liese et al. 2017 [31]	Germany	OPMD	27	NA	Surgical biopsy: oral lesions	H&E	Histopathological examination
Lassig et al. 2017 [164]	USA	OSCC	20	NA	Liquid biopsy: surgical drain fluid	Electrochemiluminescence; Patterned array; Multiplex technology	Differences in cytokine and MMP levels by disease outcomes were evaluated.
Jajodia et al. 2017 [32]	India	OSCC	48	NA	1) Brush biopsy: oral lesion<br>2) Punch biopsy: oral lesion	1) CC; LBC; AgNOR staining<br>2) H&E	1) Screening for suspected malignant oral lesions<br>2) Histopathological examination
Ishikawa et al. 2017 [210]	Japan	OC	22/66	NA	Liquid biopsy: saliva	Metabolomic analysis	Detection of markers
Grillone et al. 2017[33]	USA	OSCC	34	NA	Surgical biopsy: oral lesions	H&E	Histopathological examination
Elimairi et al. 2017 [58]	Sudan	OSCC	28	NA	Surgical biopsy: oral lesions	1) Lugol's iodine staining<br>2) NA	1) Detection of oral cancer and dysplastic lesions as well as demarcation of the extent of these lesions<br>2) Histopathological examination
da Silva et al. 2017 [34]	Brazil	OSCC/ OPMD	52	NA	1) Brush biopsy: oral lesion<br>2) Surgical biopsy: oral lesions	1) ICC; IHC<br>2) H&E; ICC; IHC	To assess the immunocytochemical and immunohistochemical correlation of adhesion (E-cadherin) and cell differentiation (involucrin) molecules in oral leukoplakia and oral squamous cell carcinoma.
Boeve et al. 2017 [85]	Netherlands	Oral maxillary cancer	11	NA	SLNB: sentinel lymph node	H&E; IHC	Histopathological examination
Angelelli et al. 2017 [35]	Italy	OC	46	NA	Surgical biopsy: oral lesions	H&E	Histopathological examination

Agarwal et al. 2016 [211]	India	OSCC	231	NA	SLNB: sentinel lymph node	NA	Histopathological examination
Buchakjian et al. 2016 [22]	USA	OSCC	NA	NA	Surgical biopsy: oral lesions	Frozen section	Tumor margin evaluation
Conway et al. 2016 [145]	UK	OPC	1212	NA	Liquid biopsy: saliva	Nucleic acid extraction; HPV genotyping;	HPV prevalence and diversity
Hettmann et al. 2016 [132]	Hungary	HNCC	NA	NA	1) Liquid biopsy: saliva<br>2) Punch biopsy: oral lesion	PCR; Sequencing	Phylogenetic Analysis
Hiraki et al. 2016 [84]	Japan	OSCC	125	cN0	SLNB: sentinel lymph node	H&E	Histopathological examination
Husso et al. 2016 [212]	Finland	OSCC	10	T1-2N0/ T2-4N0	SLNB: sentinel lymph node	Frozen section	Histopathological examination
Kaur et al. 2016 [116]	India	OC	100	NA	Brush biopsy: oral lesion	1) DNA-ICM;<br>2) May Grünwald giemsa; H&E; PAP	1) DNA ploidy analysis<br>2) Cytologic examination
Lejoy et al. 2016 [213]	India	OPMD	75	NA	Punch biopsy: oral lesion	NA	Histopathological examination
Martin et al. 2016 [140]	USA	OPMD	168	NA	Liquid biopsy: saliva	qPCR	Validation of Reference Genes for Oral Cancer Detection
Nanayakkara et al. 2016 [36]	Sri Lanka	OPMD	192	NA	1) Brush biopsy/biopsy with spatula: oral lesion<br>2) Surgical biopsy: oral lesions	1) PAP<br>2) H&E	1) Cytologic examination<br>2) Histopathological examination
Sagheb et al. 2016 [214]	Germany	TSCC	10	<T3	SLNB: sentinel lymph node	NA	Histopathological examination
Takeda et al. 2016 [59]	Japan	OSCC	35	NA	Surgical biopsy: oral lesions	RT-PCR; IHC; Immunofluorescence staining	mtDNA copy numbers and expressions of PGC-1α and TFAM
Tartaglione et al. 2016 [83]	UK	OSCC	434	cT1-T2cN0	SLNB: sentinel lymph node	H&E; IHC	Histopathological examination
Zahran et al. 2015 [141]	Arabia	OSCC/OPMD	60/100	NA	Liquid biopsy: saliva	Microarray platform and qPCR	miRNA expression analysis
Sivadasan et al. 2015 [150]	India	Healthy individuals	NA	NA	Liquid biopsy: saliva	Mass spectrometry and Proteomic analysis	Serve a reference about oral malignancies markers
Schilling et al. 2015 [82]	14 European countries	SCC	415/480	T1–T2N0	SLNB: sentinel lymph node	Frozen section and H&E	Detect metastasis
Peng et al. 2015 [103]	China	Oral/oropharyngeal carcinoma	26	cT1-2N0M0	SLNB: sentinel lymph node	1) Near-infrared imaging with ICG<br>2) Embedded in paraffin	1) Identify sentinel node<br>2) Pathologic examination
Nakamura et al. 2015 [102]	Japan	Squamous cell carcinoma of the head and neck	19	T1-T2, N0	Surgical biopsy: oral lesions<br>SLNB: sentinel lymph node	1) RI and ICG<br>2) Gamma ray probe	1) Identify sentinel node<br>2) Identified radioactivity
Mulki et al. 2015 [117]	India	OSCC	25/108	NA	1) Liquid biopsy: saliva<br>2) Brush biopsy: accessible areas	PAP	Early screening of oral cancer

Hartmann et al. 2015 [187]	Germany	OSCC	15/72	T1-T4, N1-N3	1) Oral brush biopsy: oral mucosa 2) Surgical biopsy: suspicious lesions	Melanoma-associated antigens A staining	1) Investigate the usability, specificity, sensitivity, and diagnostic accuracy of oral brush biopsy 2) As the gold standard.
Hande et al. 2015 [37]	India	OC	40	NA	Surgical biopsy: pathological lesion and the contralateral mirror image biopsy site	1) H&E 2) Immunohistochemical method for the detection of p53 antigen	1) Pathologic examination 2) Predict the altered state of oral mucosa secondary to carcinogen exposure
Graham et al. 2015 [215]	UK	Oropharyngeal carcinomas	45/57	NA	brush biopsy: oral mucosa	Dielectrophoretic method	Early identification of oral cancer in primary cancer
Den Toom et al. 2015 [81]	Netherland	OSCC	90	T1–T2, cN0	SLNB: sentinel lymph node	H&E and pan-cytokeratin antibody (AE 1/3)	Pathologic examination
De Bree et al. 2015 [38]	Netherland	HNCC	6	cN0	1) USG-FNAC: sentinel lymph node	H&E and pan-cytokeratin antibody (AE 1/3)	Better selection of lymph nodes at the highest risk of having metastases
Chinnannavar et al. 2015 [157]	India	OC	52/104	NA	Liquid biopsy: venous blood	Biochemical estimation and ninhydrin method	Diagnosis and determine the clinical stage
Yang et al. 2014 [147]	USA	OSCC	11/31	NA	1) Liquid biopsy: saliva 2) Brush biopsy: oral mucosa	Western blotting and reporter gene assays	Testing the secretory leukocyte protease inhibitor as a biomarker
Ma et al. 2014 [105]	China	OC	4/52	NA	Brush biopsy: location of the mucosal lesion	1) Feulgen staining 2) DNA-image cytometry	1) Measure the Nuclear DNA contents (ploidy) 2) Screening method for the detection of precancerous oral lesions
Gupta et al. 2014 [39]	India	Oral precancerous lesions	877	NA	1) Tongue blade and modified brush biopsy 2) Punch/surgical biopsy	1) Modified PAP 2) H&E	1) Examine dysplastic changes in the cells 2) Pathologic examination
Chianeh et al. 2014 [216]	India	OSCC	25/55	Stage 2–4	Liquid biopsy: saliva	5,5′dithiobis, 2-nitrobenzoic acid (DTNB/Ellman's reagent)	Determine the marker (Salivary protein thiols and Butyrylcholinesterase)
Schussel et al. 2013 [133]	USA	OC	30/191	NA	Liquid biopsy: saliva	Quantitative Methylation Specific PCR	Hypermethylation were associated with premalignant or malignant disease
Riaz et al. 2013 [217]	India	OPMD	50/120	NA	Surgical biopsy: the most obvious methylene blue staining area	Routine pathologic diagnosis	Examine the accuracy of the diagnostic capability of methylene blue
Mori et al. 2013 [218]	Japan	OSCC	20/47	NA	1) Brush biopsy: tongue and buccal mucosa 2) Surgical biopsy: oral lesions	1) RT-PCR 2) The electrochemical telomerase assay	1) Determine telomerase reverse transcriptase expression 2) Evaluate telomerase activity
Maurer et al. 2013 [124]	Germany	Head and neck squamous cell carcinoma	26	NA	Brush biopsy: diseased area and healthy buccal mucosa of the corresponding contralateral area	MALDI-ToF Mass Spectrometry	Early cancer diagnosis
Matthews et al. 2013 [142]	UK	OSCC	45	NA	Liquid biopsy: saliva	qPCR	DNA biomarker analysis
Kaemmerer et al. 2013 [219]	USA	OPMD	70	NA	1) Brush biopsy: mucosal lesion 2) Surgical biopsy: mucosal lesion	1) H&E and DNA-ICM examination 2) Embedded in paraffin	1) Examine tumor cells or suspicious cells and measure the Nuclear DNA contents (ploidy) 2) Pathologic examination
Graveland et al. 2013 [40]	Netherland	OPMD	23	NA	1) Brush biopsy: mucosal lesion 2) Surgical biopsy: mucosal lesion	1) p53 IHC 2) H&E and p53 IHC	1) LOH analysis 2) LOH analysis, TP53 mutation analysis and histopathological grading

Galle et al. 2013 [220]	Italy	OSCC/OPMD	103	NA	Biopsy with swabs: lesions	1) Presumptive germ tube test<br>2) PAS and GMS staining	To support the presence of Candida spp. in oral cancer and precancerous lesions
Flach et al. 2013 [221]	Nether-land	OC	285	T1-T2N0	USG-FNAC	Make cytological smear	Pathologic examination
Bianca et al. 2013 [41]	Brazil	OSCC	172	NA	1) Brush biopsy: oral lesions<br>2) Surgical biopsy: oral lesions	1) PAP<br>2) H&E	1) Cytopathological diagnosis<br>2) Pathologic examination
Cankovic et al. 2013 [222]	Serbia	OSCC	30	T1-T3, N0-N1	Biopsy: oral lesion	NA	Pathologic examination
Yoshimoto et al. 2012 [223]	Japan	Laryngeal, hypopha-ryngeal or oral cancer	177	NA	SLNB: sentinel lymph node	HE	Pathologic examination
Meric et al. 2012 [224]	Turkey	Smoker	78	NA	Punch biopsy: oral mucosa	Immunohistochemical analysis	Determine expression of p65 NF-κB, p38 MAPK, and iNOS
Melkane et al. 2012 [80]	France	OSCC	53	T1, T2 N0	SLNB: sentinel lymph node	H&E and anti-cytokeratin 22 im-munohistochemistry	Pathologic examination
MacAulay et al. 2012 [225]	Canada	OSCC/OPMD	148/369	NA	Brush biopsy: oral lesions	Modified Feulgen-Thionin staining	The amount and the distribution of DNA in the nucleus
Lohavanichbutr et al. 2012 [226]	USA	OSCC	NA	NA	Surgical biopsy: mucosal lesion	PCR	Teste Gene Expression
Kugimoto et al. 2012 [134]	Japan	OSCC	89/185	NA	Liquid biopsy: saliva	PCR	Oral cancer screening
Terada et al. 2011 [79]	Japan	OSCC	61	cT1-2, cT3	SLNB: sentinel lymph node	H&E	Pathologic examination
Saini et al. 2011 [42]	Malaysia	OSCC	105/210	NA	1) Surgical biopsy: oral lesions<br>2) Brush biopsy: oral lesions	1) H&E<br>2) PCR	1) Pathologic examination<br>2) The analysis of p53 codon 72 arginine/proline alleles
Remmerbach et al. 2011 [123]	Germany	OSCC	27/37	NA	Brush biopsy: lesion and healthy buccal aspects	Mass spectrometry	Pre-symptomatic screening detection
Paderni et al. 2011 [227]	Italy	OSCC/OPMD	175	NA	Surgical or punch biopsy: oral lesions	Direct visualization of the oral tissue autofluorescence	Early recognition and diagnosis
Kolokythas et al. 2011 [228]	USA	OSCC	NA	NA	Brush biopsy:<br>oral lesions	RT-PCR	Reproducibility of mRNA quantification
Weigum et al. 2010 [229]	USA	Visible oral lesion	41/52	NA	Brush biopsy:<br>oral lesions	Fluorescent labeling	Early detection of oral cancer
Rajput et al. 2010 [118]	India	OPMD	34/44	NA	Brush biopsy: suspicious oral le-sions	PAP and AgNOR staining	Early detection of oral cancer
Jalouli et al. 2010 [127]	Sweden	OSCC	217/423	NA	Brush biopsy: buccal mucosa or labial sulcus	PCR	Detection of EBV and HSV
Delavarian et al. 2010 [119]	Iran	OSCC/OPMD	25	NA	1) Brush biopsy: oral lesions<br>2) Surgical biopsy: oral lesions	PAP	Screening of oral premalignant and malignant lesions
Civantos et al. 2010 [78]	USA	OC	140	T1-T2, N0	SLNB: sentinel lymph node	H&E	To detect the effectiveness of SLNB
Remmerbach et al. 2009 [107]	Germany	OSCC/OPMD	47	NA	1) Brush biopsy:<br>oral lesions<br>2) Surgical biopsy: oral lesions	1) PAP, Feulgen Staining and Ag-NOR Analysis<br>2) NA	1) Multimodal cell analysis for the early detection<br>2) Pathologic examination

Hohlweg et al. 2009 [230]	Germany	OSCC	15/75	NA	1) Brush biopsy: oral lesions<br>2) Surgical biopsy: oral lesions	Histological examination	Test sensitivity and specificity of oral brush biopsy
Burns et al. 2009 [76]	Ireland	OSCC	13	T1–T3N0	SLNB: sentinel lymph node	H&E and cytokeratin staining	Evaluate metastatic disease
Atula et al. 2009 [77]	UK	OSCC	107	NA	SLNB: sentinel lymph node	H&E and AE1/AE3 staining	Pathologic examination
Terada et al. 2008 [104]	Japan	OC	44	NA	SLNB: sentinel lymph node	HE, MGG and PAP	Pathologic examination
Santaolalla et al. 2008 [75]	Spain	OSCC	22	T1T2T3cN0	SLNB: sentinel lymph node	H&E, cytokeratin AE1/AE3 staining	Pathologic examination
Sanjay et al. 2008 [231]	India	OSCC	30/60	NA	Liquid biopsy: saliva	Biochemical analysis of saliva	Early detection of cancer
Navone et al. 2008 [232]	Italy	OSCC/OPMD	164	NA	1) Brush biopsy(curette): oral lesions<br>2) Surgical biopsy: oral lesions	Processed histologically	To value the accuracy of micro-biopsies
Matsuzuka et al. 2008 [71]	Japan	OSCC	10	NA	SLNB: sentinel lymph node	H&E	Measure the area of malignant tumor and that of resid-ual normal<br>lymph tissue
Keski et al. 2008 [72]	Finland	OSCC	13	pT1N0	SLNB: sentinel lymph node	H&E and cytokeratin AE1/AE3	Evaluate micrometastasis
Chone et al. 2008 [73]	Brazil	HNCC	35	cN0	SLNB: sentinel lymph node	H&E	Pathologic examination to test occult metastasis
Bilde et al. 2008 [74]	Denmark	OSCC	51	T1T2N0M0	SLNB: sentinel lymph node	H&E and cytokeratin AE1/AE3	Pathologic examination to determine lymph node me-tastases
Vigili et al. 2007 [69]	Italy	OSCC	12	cT1-T2N0	SLNB: sentinel lymph node	H&E and cytokeratin AE1/AE3	Immunohistochemistry analysis
Upile et al. 2007 [179]	UK	OSCC	40	T stage I/II	Surgical biopsy: oral lesions	Methylene blue and microendoscope	Determination of surgical margins
Thomsen et al. 2007 [70]	Denmark	OSCC	40	T1T2 cN0	SLNB: sentinel lymph node	H&E and cytokeratin	Pathologic examination
Sandro J Stoeckli et al. 2007 [68]	Switzer-land	OSCC	79	T1T2	SLNB: sentinel lymph node	H&E	Pathologic examination to assess the feasibility of SLNB
Majumder et al. 2007 [135]	India	OSCC	310/923	NA	Liquid biopsy: blood	PCR	Determined genotypes
Hirshberg et al. 2007 [233]	Israel	OSCC/OPMD	29/54	NA	1) Brush biopsy: oral lesions and opposite normal site<br>2) Surgical biopsy: oral lesions	1) H&E<br>2) MGG and I-FISH experiments	1) Pathologic examination<br>2) Detect non-diploid cells to enhance early detection
Gupta et al. 2007 [43]	India	OPMD	96	NA	1) Brush biopsy: oral lesions<br>2) Surgical biopsy: oral lesions	1) PAP<br>2) H&E	Evaluate the usefulness of toluidine blue and brush bi-opsy
Chen et al. 2007 [234]	China	OPMD	58	NA	Surgical biopsy: oral lesions	NA	Pathologic diagnosis

Terada et al. 2006 [67]	Japan	OC	15	N0	SLNB: sentinel lymph node	H&E	Pathologic diagnosis
Songra et al. 2006 [235]	UK	OSCC	26	NA	Surgical biopsy: oral lesions	Ultrasound scanning and histology	Evaluate the usefulness of ultrasound imaging
Poh et al. 2006 [236]	Canada	OSCC	20	T0-T2/ stage 0-II	Punch biopsies: the tumor and tumor margins	NA	Pathologic diagnosis
Maraki et al. 2006 [106]	Germany	OPMD	58	NA	1) Brush biopsy(curette): oral lesions 2) Surgical biopsy: oral lesions	PAP; Feulgen Staining	Measurement of DNA contents and pathologic diagnosis
Kujan et al. 2006 [237]	UK	Healthy volunteer	50	NA	Brush biopsy: buccal mucosa and lateral border of tongue	FHIT immunocytochemistry staining	Early detection of cancer and precancer
Kovacs et al.2006 [61]	Germany	OSCC	77	T1–4aN0	SLNB: sentinel node	H&E; cytokeratin AE1/AE3	Pathologic diagnosis
Khafif et al. 2006 [238]	Israel	OSCC	20	T1-4N0	SLNB: sentinel node	NA	Pathologic diagnosis; Immunohistochemistry examine
Gabriel et al. 2006 [156]	USA	Healthy volunteer	56	NA	1) Liquid biopsy: blood, urine 2) Brush biopsy: oral lesions	1) High-performance liquid chromatography analysis 2) Sigma staining	Examine the concentration of carotenoids, retinoids, and tocopherols
Civantos et al. 2006 [62]	USA	Oral and cutaneous malignancy	106	T1 to T3	SLNB: sentinel node	H&E	Pathologic diagnosis
Bilde et al. 2006 [63]	Denmark	OSCC	34	stage I and II (T1-2N0M0)	SLNB: sentinel node	Lymphoscintigraphic H&E and cytokeratin AE1/AE3	Provide accurate information and Pathologic diagnosis
Thomsen et al. 2005 [64]	Denmark	OSCC	30	T1 T2	SLNB: sentinel node	H&E and cytokeratin AE1/AE3	Pathologic diagnosis
Thomsen et al. 2005 [65]	Denmark	OSCC	40	T1 T2	SLNB: sentinel node	H&E and cytokeratin (CK 1)	Pathologic diagnosis
Thomsen et al. 2005 [66]	Denmark	OSCC	40	T1/T2 N0	SLNB: sentinel node	H&E and cytokeratin (CK-KL1)	Pathologic diagnosis
Ram et al. 2005 [44]	Malaysia	OSCC	40	NA	Surgical biopsy: oral lesions	H&E	Pathologic diagnosis
Myo et al. 2005 [239]	Japan	OSCC	45	Stage I and II (T1-2N0M0)	Fine needle aspiration biopsy	FISH analysis	To evaluate the value of cyclin D1 gene numerical aberrations
Minamikawa et al. 2005 [240]	Japan	OSCC	632	NA	SLNB: sentinel node	Pathologic analysis	Evaluate the levels of metastatic lymph nodes
Hsu et al. 2005 [241]	USA	OC	NA	NA	Surgical biopsy: oral lesions	Molecular-specific fluorescent contrast; Agent and single-wavelength spectroscopy	Detection of the Molecular Changes
Fischer et al. 2005 [242]	USA	Upper aerodigestive tract lesions	75	NA	1) Punch biopsy: oral lesion 2) Surgical biopsy: oral lesions	Staining	Pathologic examination
Sokolov et al. 2004 [45]	USA	OPMD	25	NA	Surgical biopsy: oral lesions	H&E	Histopathological examination
Seoane et al. 2004 [46]	Spain	NA	354	NA	Surgical biopsy: oral lesions	H&E	Clinical and pathological diagnoses

Maraki et al. 2004 [120]	Germany	OC	98	NA	1) Brush biopsy: oral suspicious lesions<br>2) Surgical biopsy: oral lesions	1) PAP; Detection of malignant cells<br>2) NA	1) Cytological diagnoses; detect malignant cells<br>2) Histopathological examination
Hamakawa et al. 2004 [243]	Japan	OC	10	T1/T2	SLNB: sentinel node	RT-PCR	Morphological diagnosis; Genetic diagnosis
Chikamatsu et al. 2004 [60]	Japan	OSCC	11	T2T3 N0N1N2bN2c	SLNB: sentinel node	H&E	Examine for lymph node involvement due to tumor.
Beevi et al. 2004 [47]	India	OSCC	15/30	clinical stage III/IV	1) Liquid biopsy: blood<br>2) Punch biopsy: oral mucosa	1) Biochemical Measurements<br>2) H&E	1) To provide evidence of the relationship between lipid peroxidation and oral cavity cancer<br>2) Histopathology examination
Remmerbach et al. 2003 [121]	Germany	OSCC	53/75	NA	Brush biopsy: suspicious or pathological mucosal areas	PAP and AgNOR-staining	Cytopathological diagnosis and the number of AgNOR dots per nucleus distinguishing
Epstein et al. 2003 [244]	Canada	Upper aerodigestive tract cancer	30/668	NA	1) Liquid biopsy: saliva<br>2) Punch biopsy: suspicious lesions	1) Tolonium chloride staining<br>2) NA	1) Decide whether lesion require an urgent biopsy<br>2) Pathologic examination
Werner et al. 2002 [245]	Germany	The upper aerodigestive tract SCC	48	N0N1	SLNB: sentinel node	NA	Pathologic examination
Weinstein et al. 2002 [136]	USA	Oral and pharynx cancer	519	NA	1) Liquid biopsy: blood, urine<br>2) Brush biopsy: mucosal surface	PCR and Laboratory analyses (Homocysteine and Methylenetetrahydrofolate Reductase)	Examined the relationships between serum homocysteine levels and methylenetetrahydrofolate reductase (MTHFR) C677T polymorphism genotype
McCullough et al. 2002 [48]	UK	OSCC/OPMD	223	NA	Surgical biopsy: suspicious lesions	PAS and H&E	Assess the presence of yeast and Pathologic examination
da Costa et al. 2002 [49]	Brazil	Healthy individual	11	NA	Surgical biopsy: lingual gingival margin of the right or left second upper premolar	H&E and PCNA	Pathologic examination
Betz et al. 2002 [246]	Germany	OSCC/OPMD	85	NA	Surgical biopsy: the site of the suspected or proven malignancy	NA	Pathologic examination
Nunes et al. 2000 [128]	Brazil	OSCC	19/29	NA	Brush biopsy: brush the tumor	PCR	The detection of tumor DNA
Kusukawa et al. 2000 [143]	Japan	OSCC	20	T1-T4	1) Surgical biopsy: oral lesions<br>2) Liquid biopsy: blood	1) NA<br>2) RT-PCR	1) Pathologic examination<br>2) Detection of microsatellite allele loss
Harty et al. 2000 [129]	USA	OSCC	219/367	NA	Brush biopsy: oral mucosa and tongue	PCR	Assess the DNA quality
Hall et al. 2000 [189]	UK	NA	52	NA	Surgical biopsy: oral lesions	1) Trypan blue exclusion assay<br>2) FACS analysis	To assess viability measure the range in variation in fluid phase endocytic capability
Sciubba et al.1999 [50]	USA	OSCC/OPMD	945	NA	1) Brush biopsy: OralCDx specimens<br>2) Surgical biopsy: oral lesions	1) PAP; Stained slides then were scanned by the OralCDx computer system<br>2) H&E	1) Detection of innocuous-appearing oral cancers at early.<br>2) Histopathological examination
Epstein et al. 1997 [247]	UK	OSCC	81	NA	Surgical biopsy: oral lesions	NA	Histologic examination
Erenmemisoglu et al. 1995 [51]	Turkey	Tobacco users	80	NA	Surgical biopsy: buccal mucosa	1) Cytological smears<br>2) H&E	Early detection

Wood et al. 1994 [52]	USA	Oral leukoplakia	12/24	NA	Punch biopsy: oral lesions	1) IHC 2) H&E	1) Accumulation of p53 protein was assessed 2) Histologic examination;
Cox et al. 1993 [248]	UK	OSCC	16/21	NA	Surgical biopsy: oral lesions	DNA extraction; hybridization reconstruction test; probe removal and re-use of DNA blot	Detection of HSV-1 and HPV type 16 DNA sequences
Bahr et al. 1992 [23]	Germany	OC	24	NA	Surgical biopsy: oral lesions	Frozen section	Detection of tumor resection borders
Migliorati et al. 1986 [53]	USA	OPMD	20	NA	1) Punch biopsy: oral lesions 2) Liquid biopsy: blood	1) H&E 2) ABC-immunoperoxidase technique	1) Histologic examination 2) Phenotypic identification of mononuciear cells
Abdulkader et al. 1981 [249]	Malaysia	OC	60	NA	Punch biopsy: oral lesions	1) Saline extract 2) Immuno-electrophoresis	Demonstration of a tumour associated antigen
Benson et al. 1975 [250]	USA	OC	73	NA	Surgical biopsy: oral lesions	NA	Foretelling lesion outcome
Dunn et al. 1972 [251]	NA	Laryngeal/pharyngeal/OC	44	NA	1) Surgical biopsy: oral lesions 2) Liquid biopsy: blood	NA	1) Histopathology of lesions examined T 2) Testing for serum tetracycline
Pindborg et al. 1971 [54]	India	OC	10169	NA	Punch biopsy: buccal mucosa	H&E	Histologic examination
Pindborg et al. 1967 [55]	India	OC/ OSMF	100	NA	Surgical biopsy: oral lesions lesions	H&E	Histologic examination
Glucksmann et al. 1967 [56]	UK	Buccal carcinoma	172	NA	Surgical biopsy: oral lesions	H&E	The value of histological prognosis.

Abbreviations: OSCC: oral squamous cell carcinoma; OC: oral cancer; H&E: hematoxylin and eosin staining; OSMF: oral submucous fibrosis; ATR-FTIR: attenuated total reflection fourier transform infrared spectroscopy; OPMD: oral potentially malignant disorders; NA: no information given in the article; VIA: visual inspection acetic acid; USA: the United States of America; VILI: visual inspection with lugol's iodine; DNA-ICM: DNA-image cytometry; SSOCT: swept source optical coherence tomography; PAP: papanicolaou stain; MGG: may Grunwald Giemsa; FT-IR: fourier transform infrared spectroscopic techniques; SCCT: squamous cell carcinoma of the tongue; ddPCR: Droplet Digital polymerase chain reaction; OCSCC: oral cavity squamous cell carcinoma; LC-MS/MS: liquid chromatography and tandem mass spectrometry; ELISA: enzyme linked immunosorbent assay; FNAC: ultrasound-guided fine-needle aspiration cytology; IHC: immunohistochemistry; OSCG: oral squamous carcinoma group; MBB: modified brush biopsy; LBC: liquid-based cytology; OPC: oropharyngeal cancer; OLK: oral leukoplakia; OLBC: oral liquid-based brush cytology; SLS: synchronous luminescence spectroscopy; ED: epithelial dysplasia; HNSCC: head and neck cancer squamous cell carcinoma; RT-PCR: real-time polymerase chain reaction; FEP: fibro-epithelial polyps; HTS: high-throughput nucleotide sequencing; BChE: enzyme-butyryl cholinesterase; CC: conventional cytology; LBC: liquid-based cytology; ICC: immunocytochemistry; HPV: human papilloma virus; UK: United Kingdom; TSCC: squamous cell carcinoma of the tongue; PGC-1α: peroxisome proliferator-activated receptor gamma coactivator-1 alpha; TFAM: mitochondrial transcription factor A; ICC: immunocytochemistry PMD: potentially malignant disorders; CEUS-CNB: contrast-enhanced ultrasound guided transoral core needle; ICG: indocyanine green; RI: radioisotope; USG-FNAC: ultrasound-guided fine-needle aspiration cytology; LOH: loss of heterozygosity; PAS: periodic acid-schiff ; GMS: grocott's methenamine silver; AgNOR: silver stained nucleolar organizer regions; PCNA: proliferating cell nuclear antigen; MALDI-ToF MS: matrix-assisted laser desorption/ionisation-time of flight mass spectrometry; FACS: Fluorescence activated cell scanning; [a] If the marker appears in the table, it will correspond to the following content. [b] This is a country where the patient is enrolled. [c] When only one figure is available, it means the total number of cases. * Solid biopsy included in punch biopsy, incisional biopsy and excisional biopsy. **Oral lesions include tumor tissue, suspected lesions and other lesions associated with oral cancer.
